# Sorafenib, a multikinase inhibitor, induces formation of stress granules in hepatocarcinoma cells

**DOI:** 10.18632/oncotarget.5980

**Published:** 2015-11-02

**Authors:** Pauline Adjibade, Valérie Grenier St-Sauveur, Miguel Quevillon Huberdeau, Marie-Josée Fournier, Andreanne Savard, Laetitia Coudert, Edouard W. Khandjian, Rachid Mazroui

**Affiliations:** ^1^ Centre de Recherche du toCHU de Québec-CHUL, Université Laval, Québec, PQ, Canada; ^2^ Département de Biologie Moléculaire, Biochimie Médicale et Pathologie, Faculté de Médecine, Université Laval, Québec, PQ, Canada; ^3^ Centre de Recherche en Cancérologie de L'Université Laval, Québec, PQ, Canada; ^4^ Centre de Recherche, Institut Universitaire en Santé Mentale de Québec, PQ, Canada; ^5^ Département de Psychiatrie et de Neurosciences, Faculté de Médecine, Université Laval, Québec, PQ, Canada

**Keywords:** stress granules, sorafenib, PERK, eIF2a, ATF4

## Abstract

Stress granules (SGs) are cytoplasmic RNA multimeric bodies that form under stress conditions known to inhibit translation initiation. In most reported stress cases, the formation of SGs was associated with the cell recovery from stress and survival. In cells derived from cancer, SGs formation was shown to promote resistance to either proteasome inhibitors or 5-Fluorouracil used as chemotherapeutic agents. Despite these studies, the induction of SGs by chemotherapeutic drugs contributing to cancer cells resistance is still understudied. Here we identified sorafenib, a tyrosine kinase inhibitor used to treat hepatocarcinoma, as a potent chemotherapeutic inducer of SGs. The formation of SGs in sorafenib-treated hepatocarcionoma cells correlates with inhibition of translation initiation; both events requiring the phosphorylation of the translation initiation factor eIF2α. Further characterisation of the mechanism of sorafenib-induced SGs revealed PERK as the main eIF2α kinase responsible for SGs formation. Depletion experiments support the implication of PERK-eIF2α-SGs pathway in hepatocarcinoma cells resistance to sorafenib. This study also suggests the existence of an unexpected complex regulatory balance between SGs and phospho-eIF2α where SGs dampen the activation of the phospho-eIF2α-downstream ATF4 cell death pathway.

## INTRODUCTION

When exposed to environmental stresses, cells activate pathways that induce a coordinated response of mRNA translation and turnover that protects cells from stress-induced damage and promotes their survival. One such stress pathway involves the formation of stress granules (SGs), which are cytoplasmic bodies induced by various stimuli involved in cancer treatments such as ionizing radiation [1], hypoxia [2], and proteasome inhibitors [3, 4, 5]. Since these stresses are all known to inhibit translation initiation, SGs are thought to represent sites of repression of the translation of specific mRNAs.

SGs contain small ribosomal subunit, translation initiation factors, mRNA with associated RNA binding proteins, and signaling molecules [6]. Sequestration of signaling molecules such as Rack1 [7], Traf2 [8], and Raptor [9] in SGs inhibits stress-mediated apoptotic pathways in tumor cells. SGs also promote tumor cell survival upon either radiotherapy [1] or chemotherapy [3, 4, 10, 11] by sequestering and preventing the degradation of mRNAs encoding key growth factors and antiapoptotic proteins including VEGF and p21. Targeting SGs-inducing pathways may thus constitute a novel chemotherapeutic approach.

The inhibition of translation initiation is a key event that triggers SGs formation [12]. Phosphorylation of eIF2α at serine 51 is a well characterised pathway known to inhibit translation initiation in response to various stressful conditions including hypoxia [13], viral infection [14], oxidative and Endoplasmic Reticulum (ER) stresses [15]. Phosphorylation of eIF2α inhibits translation initiation by stalling translation initiation complexes in an inactive form, whose accumulation results in SGs formation [16]. However, phosphorylation of eIF2α was shown to either promote or impede stress-mediated apoptotic pathways [12]. The proapoptotic role of phosphorylated eIF2α is attributed to the translational down-regulation of survival proteins such as bcl-x [17]. Phospho (P)-eIF2α promotes cell death by inducing preferential translation of ATF4 [18, 19, 20], a transcription factor known to activate transcription of key stress apoptotic factors such as ATF3 and CHOP [18, 19, 20]. Downregulation of ATF4 was however shown to prevent cancer cells resistance to anticancer drugs [21], indicating that a minimal expression of ATF4, which is driven by P-eIF2α is nevertheless required for cancer cells survival to chemotherapeutics. This chemoresistant role of ATF4 was attributed to its activity in driving the expression of antioxidant and chapronnes genes that favor cell survival and growth [18]. The survival properties of phosphorylated eIF2α are mediated by additional multiple mechanisms including the activation of the PI3K-PKB-mTOR signaling pathway [22], the induction of transcription factor NF-kB [20], and the formation of SGs [4, 23, 24, 25].

eIF2α phosphorylation-mediated SGs formation involves the activation of one of the four stress specific eIF2α kinases. While the SGs-inducing effect of oxidative stress is mediated by HRI [26], viral infection seems to trigger SGs formation through PKR activation [27, 28]. GCN2 is the main eIF2α kinase that drives SGs formation under conditions of amino acid deprivation [29] and PERK is responsible for eIF2α phosphorylation and associated SGs formation as a result of the accumulation of unfolded proteins during ER stress [30]. Despite the well-established role of eIF2α kinases in regulating the cellular stress response, their role in cancer was neglected. Recent studies now implicated the activity of GCN2, HRI and PERK in cancer biology [31]. In particular, PERK has now emerged as a potential factor that promotes tumor growth and angiogenesis [32, 33], which is consistent with its high activity observed in human tumors including breast and glioma [34]. Activated PERK was also implicated in cancer cells resistance to anticancer drugs [34, 35].

Sorafenib (Nexavar^®^), an Raf1/Mek/Erk kinase inhibitor approved for advanced hepatocarcinoma cells (HCC) [35], was recently shown to induce an ER stress characterised by the activation of PERK-phosphorylation of eIF2α axis in leukemia cells [36]. We report here that sorafenib is a potent inducer of SGs in cancer cells of various origins, including breast, prostate, cervix, and HCC. We focused on characterising sorafenib-induced SGs in HCC, the clinical targets of sorafenib [36]. Formation of SGs in sorafenib-treated HCC requires the phosphorylation of the translation initiation factor eIF2α through the stress kinase PERK. However, differential formation of SGs was observed between HCC. While SGs were efficiently induced in sorafenib-resistant HCC (Hep3B), they were significantly reduced in the less resistant HCC (Huh-7) cells. This differential formation of SGs was inversely correlated with the expression of ATF4, the downstream target of P-eIF2α. Supporting data of the role of SGs in buffering ATF4 translation during sorafenib exposure is provided by FISH experiments showing the localisation of ATF4 mRNA in SGs, thereby preventing its association with translating polysomes and keeping its expression minimal. This minimal expression of ATF4 in SGs-forming HCC is relevant because its elimination by siRNAs prevented their survival. Similar results were obtained by disrupting SGs through PERK depletion. Our study suggests that PERK-mediated SGs formation contribute to HCC resistance to sorafenib, in part by modulating ATF4 expression.

## RESULTS

### Sorafenib induces SGs formation in various cancer cells including HCC

**Figure 1:**
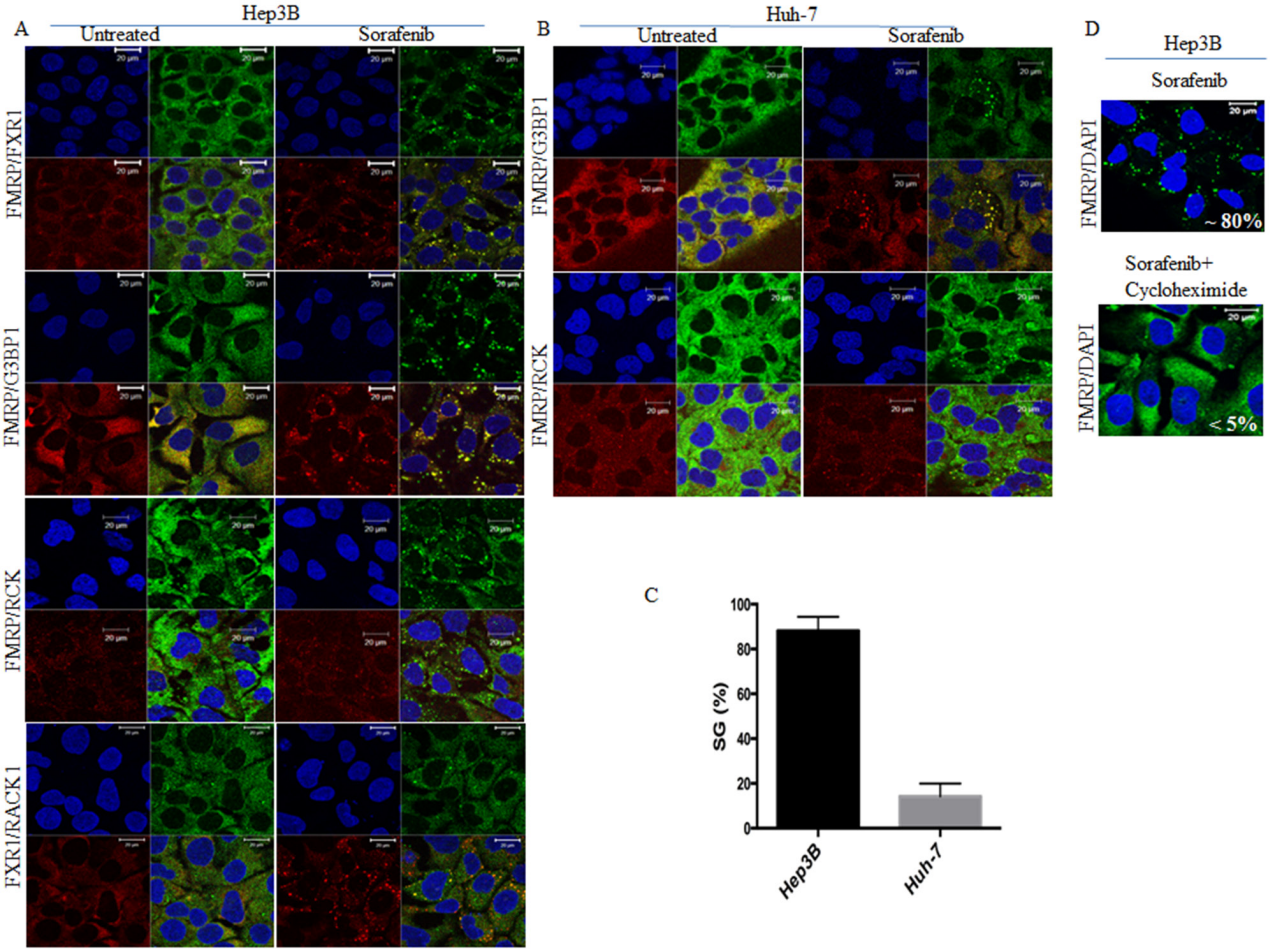
Sorafenib induces SGs in HCC Hep3B and Huh-7 cells were treated with sorafenib (10 μM) for two hours or left untreated. **A–C.** Hep3B (A) and Huh-7 (B) were processed for immunofluorescence to detect SGs using antibodies specific to several SGs markers (FMRP, G3BP1, FXR1 and RACK1), or to P-bodies (RCK). Blue DAPI staining depicts nuclei. Scale bars are shown. The percentage of cells harboring SGs (>3 granules/cell) is indicated in (C). These representative results are from 50 different fields and 10 different experiments containing a total of 5000 cells. **D.** Hep3B were treated with both sorafenib (10 μM) and cycloheximide (100 μg / ml) for two hours and then were processed for immunofluorescence as above. DAPI stains for nuclei. The percentage of SGs-positive cells is indicated.

To determine if sorafenib induces SGs formation, we treated various cancer cell lines with 5–25 μM of sorafenib and assessed SGs formation by immunofluorescence using antibodies specific to several canonical SGs markers. We found that sorafenib potently (~ 80%) induces SGs in various cancer cells including HeLa (cervix), MCF-7 (breast), PC3 and LnCaP (prostate) (Fig. S1), and Hep3B (HCC) cell lines (Fig. 1). We focused our study on characterising sorafenib-SGs in HCC. Dose-response experiments show that the minimal concentration of sorafenib that induces SGs in a maximum (>80%) of Hep3B cells at 2 hours of treatment is 10 μM. This concentration is physiological relevant since it corresponds to the average plasma concentrations measured in patients receiving sorafenib, i.e., 5–10 μM [37]. Time course analysis of SGs formation upon treatment of Hep3B with 10 μM sorafenib shows that SGs started forming as soon as 30 minutes (data not shown), picked at 2 h (>80%) following treatment (Fig. 1A and 1C), and lasted over 8 h post-treatment (Fig. S2A). The number of SGs decreases however over time to reach ~ 20% at 24 h post-treatment (data not shown). SGs induction by sorafenib in HCC is not restricted to Hep3B since they also form in sorafenib-treated Huh-7 (Figs. 1B–1C, and S2A), albeit at much less efficiency (10–20%), as compared to Hep3B (>80%) (Fig, 1C). Sorafenib-induced SGs in HCC contain canonical SGs markers including FMRP, FXR1, G3BP1 (Fig. 1), eIF4E and eIF4GI (Fig. S3A), as well as poly(A)+ mRNA (Fig. S4). RACK1, a component of arsenite-induced SGs [38], was also found to partially localise in SGs induced by sorafenib (Fig. 1A–1B). Sorafenib-induced SGs lack both RCK (Fig. 1A and 1B) and GFP-dcp1a (Fig. S3B), two classical components of the cytoplasmic mRNA decay bodies (P-bodies) [39], indicating that sorafenib-induced SGs are distinct from P-bodies. Moreover, sorafenib does not affect the formation of RCK-containing P-bodies in HCC, indicating a specific effect of sorafenib on SGs. As expected, treatment of HCC with the SGs inhibitor cycloheximide [40] disrupts sorafenib-SGs (Fig. 1D). Altogether, these results show that treatment of HCC with sorafenib induces classical SGs.

### Sorafenib induces SGs in an eIF2α phosphorylation-dependent manner

**Figure 2:**
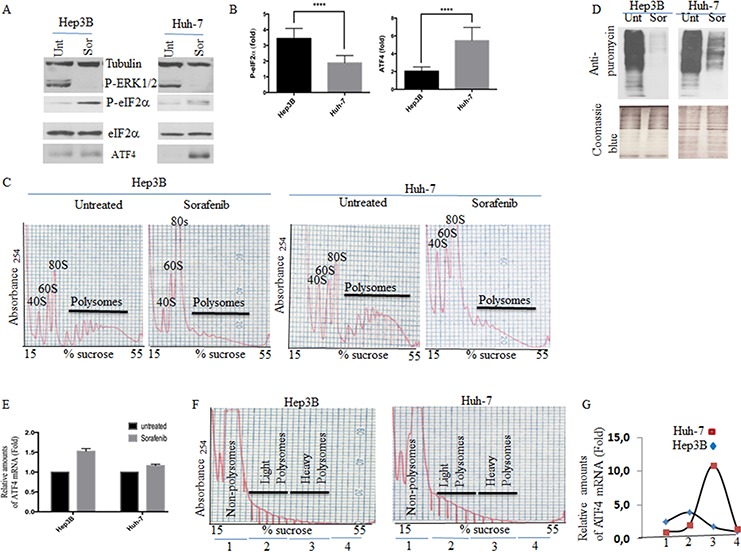

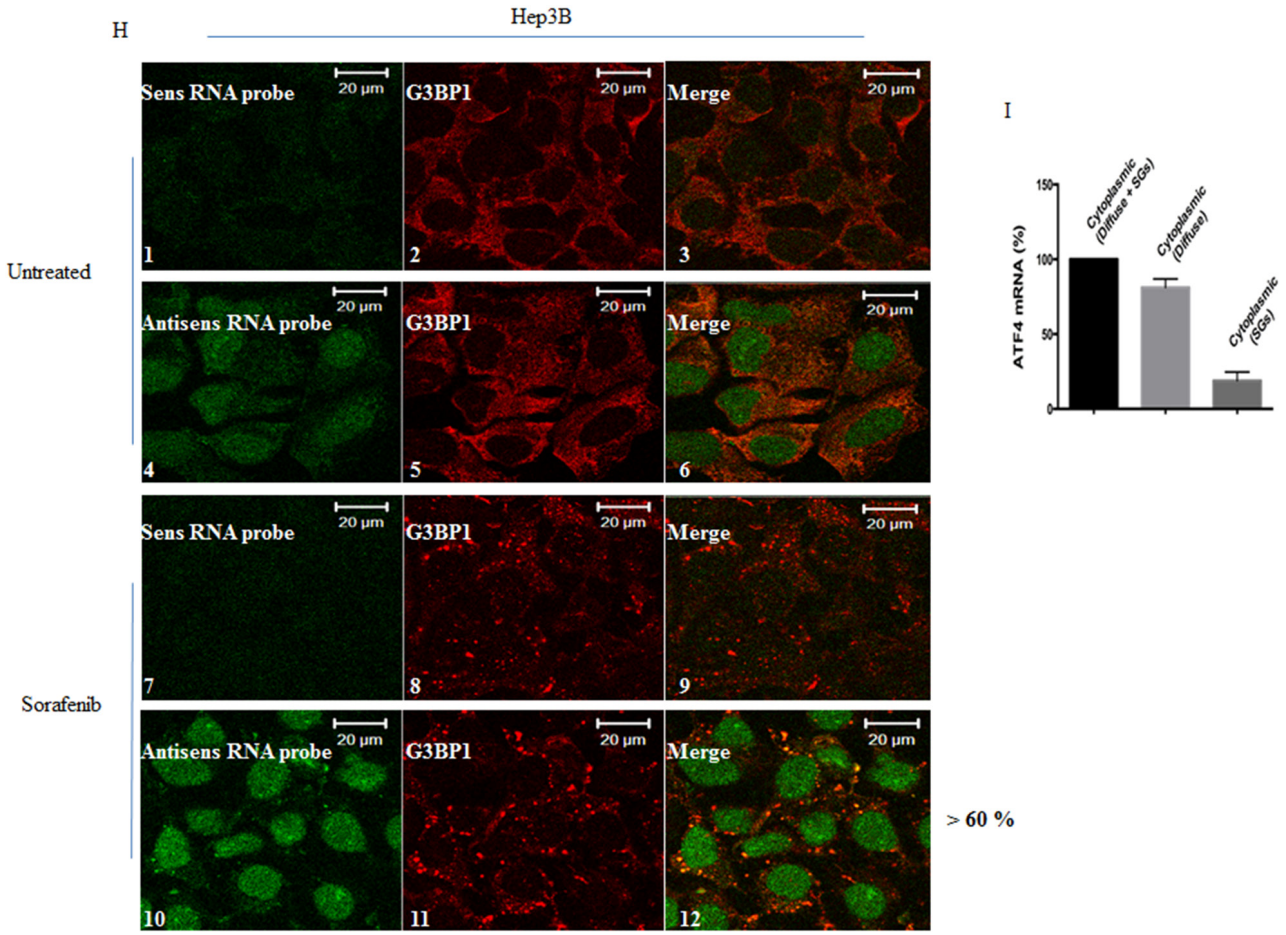
Analysis of eIF2α phosphorylation, ATF4 mRNA expression and localisation, and general translation initiation in sorafenib-treated HCC **A–D.** Hep3B and Huh-7 were treated with sorafenib for two hours and then collected. (A) Protein content was analysed for the expression of phospho-eIF2α, phospho-ERK1/2, and ATF4 by western blot using specific antibodies. Tubulin and eIF2α serve as loading controls. (B) The amounts of phosphorylated eIF2α and ATF4 were determined by densitometry quantitation of the film signal using Image Studio™ Lite Software, normalised against total eIF2α and expressed as indicated. ****P < 0.0001 (Student's *t*-test). The results are representative of more than 5 different experiments. (C) Cytoplasmic extracts were prepared and fractionated on sucrose gradients. The indicated polysome profiles were monitored by measuring the OD_254_. (D) Hep3B and Huh-7 were treated with sorafenib for one hour and fifty minutes, then puromycin (50 μg/ml) was added for an additional ten minutes. Cells were collected and protein content was analysed by western blot for puromycin incorporation into nascent polypeptide chains using anti-puromycin antibodies (top panel). Coomassie Blue (bottom panel) staining shows equal protein loading. **E.** RNA content was isolated and the amount of ATF4 mRNA relative to Actin mRNA was quantified by real-time q(RT)-PCR using the ΔΔCt method. The results are presented as the mean of triplicate measurements, with error bars corresponding to the SEM. **F–G.** Cytoplasmic extracts of sorafenib-treated Hep3B and Huh-7 were prepared, fractionated on sucrose gradients and their polysomes profiles recorded (F) as above. (G) RNA content was isolated from pooled non-translating monosomal (pool 1), low translating polysomal (light polysomes; pool 2) and high translating polysomal (heavy polysomes; pool 3) fractions and analysed for levels of ATF4 mRNA by qRT-PCR using the ΔΔCt method. ATF4 mRNA levels were standardised against 18s ribosomal RNA and expressed as a percentage of total RNA. (G) The results are representatives of two independent experiments. **H–I.** FISH experiments. (H) untreated or sorafenib-treated Hep3B cells were fixed, permeabilised, and then incubated with 3 nM of an Alexa Fluor 488-labeled antisense RNA probe to detect ATF4 mRNA (panels 4 and 10) or with the Alexa Fluor 488-labeled sense probe as control (panels 1 and 7). SGs were detected using anti-G3BP1 antibodies (red signal). Merged pictures show a clear localisation of ATF4 mRNA signal in SGs. The percentage of cells harboring SGs positive for ATF4 mRNA is indicated at the right of panel 12. Shown are typical results from three different fields and two different experiments containing a total of 1000 cells. (I) Quantification of FISH signal. Densitometry quantification of FISH signal with Adobe Photoshop software. Pixels numbers and mean intensities were recorded for the selected regions (SGs, diffuse cytoplasm and background). The mean intensity was multiplied by the number of pixels for the region selected in order to obtain the absolute intensity. The absolute intensity of the background region was subtracted from each region of interest. Relative intensities were then calculated and correspond to the absolute intensities normalised to the absolute intensity of the reference.

The formation of stress-induced classical SGs involves multiple pathways [41], the main being the phosphorylation of eIF2α. We thus sought to further characterise the formation of sorafenib-SGs in HCC by assessing if it requires the phosphorylation of eIF2α. First, we monitored the phosphorylation of eIF2α in both sorafenib-treated Hep3B and Huh-7 cells. Control experiments show that sorafenib inhibits the autophosphorylation activity of its MEK/ERK targets [42], similarly in Hep3B and Huh-7 (Fig. 2A). This indicates that sorafenib is effective in both cell lines. Sorafenib treatment induces phosphorylation of eIF2α in both Hep3B and Huh-7 (Figs. 2A and S2B), which correlates with formation of SGs (Figs. 1A–1B and S2A). As shown in Fig. 2A–2B (see also Fig. S2B), the phosphorylation of eIF2α in sorafenib-treated Hep3B is however significantly more induced (>3 fold) than in sorafenib-treated Huh-7 (~ 1.5 fold), which as described earlier are less prone to form SGs than Hep3B (Figs. 1A–1B and S2A).

While phosphorylation of eIF2α triggers the general inhibition of translation initiation upon stress, it allows the preferential translation of specific mRNAs whose products either promote or prevent stress-induced cell death [15]. ATF4 mRNA is the main mRNA to be translated specifically when eIF2α is phosphorylated [15]. We thus tested if sorafenib-induced phosphorylation of eIF2α in HCC is sufficient to trigger the inhibition of translation initiation, thus allowing the preferential expression of ATF4. Analysis of polyribosomes profiles confirms that translation initiation was efficiently blocked in both Hep3B and Huh-7, following treatment with sorafenib (Fig. 2C). We corroborated these results by both metabolic labeling (Fig. S2C) and ribopuromycylation (Fig. 2D) [43], showing a net reduction of general translation in both cell lines upon treatment with the drug. Thus, sorafenib treatment inhibits translation initiation in HCC, most-likely through phosphorylation of eIF2α.

As expected, the expression of ATF4, the downstream target of P-eIF2α, was also induced in HCC upon sorafenib treatment. The induction of ATF4 expression occurs however with different efficiencies between sorafenib-treated Hep3B and Huh-7. Unexpectedly, ATF4 was highly induced (~ 5.5 fold) in Huh-7, as compared to Hep3B (<2 fold) (Figs. 2A–2B and S2B). This is surprising since phosphorylation of eIF2α, which is responsible for the expression of ATF4, is significantly lower in Huh-7 as compared to Hep3B. The steady state level of ATF4 mRNA is similar between Hep3B and Huh-7 treated with sorafenib (Fig. 2E), indicating that the differential expression of ATF4 observed between SGs-forming Hep3B and SGs-deficient Huh-7 is likely to occur posttranscriptionally.

Using sucrose gradient polyribosomes fractionation assays, we found that ATF4 mRNA is highly enriched in fractions corresponding to high translating polysomes (heavy polysomes; pool 3) as compared to either non-translating monosomes (pool 1) or to low translating polysomes (light polysomes; pool 2) in sorafenib-treated Huh-7 (Fig. 2F–2G). This result is consistent with the high expression of ATF4 mRNA observed in sorafenib-treated Huh-7 (Figs. 2A–2B and S2). In contrary, ATF4 mRNA was barely detected in the high translating polysomes of sorafenib-treated Hep3B (Fig. 2F–2G), which is consistent with the assumption that is repressed in this cell line upon sorafenib treatment. Because the association of specific mRNAs with SGs prevents their expression, [10, 29, 44], we anticipated the possibility that ATF4 mRNA may be trapped in SGs, which would then dampen its overproduction in Hep3B.

Using FISH experiments, we found that indeed ATF4 mRNA is localised in SGs (Fig. 2H; lanes 10 and 12). Quantification of ATF4 mRNA FISH signal (Fig. 2I) shows that ~20% of the cytoplasmic ATF4 mRNA is trapped in SGs. This quantification is likely to be underestimated because it does not take into account any mobile fraction of this mRNA that transiently associates with SGs, therefore precluding its detection by FISH in fixed cells (see discussion). Nevertheless, our data suggest that at least a sub-fraction of ATF4 mRNA produced in sorafenib-treated Hep3B is stably trapped and repressed in SGs. Together, these results show that sorafenib treatment differentially induces SGs formation, phosphorylation of eIF2α and ATF4 expression in HCC.

**Figure 3:**
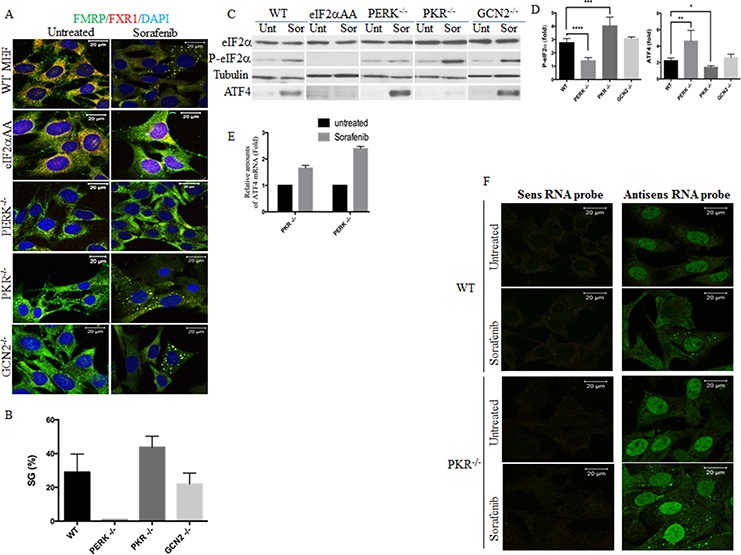
Sorafenib induces SGs in MEFs The indicated MEFs were treated with 25 μM of sorafenib (Sor) for two hours or left untreated (Unt). **A–B.** Cells were fixed and SGs were visualised by immunofluoresence using anti-FMRP and anti-FXR1 antibodies. Shown are merge pictures. Blue staining depicts nuclei. Representative results from 5 different fields and 4 different experiments containing a total of 1000 cells are shown. The percentage of cells harboring SGs (> 3 granules/cell) is indicated in (B). Scale bars are shown. **C–D.** Protein content of collected cells was analysed by western blot (C) for the amount of P-eIF2α and ATF4 using specific antibodies. eIF2α and tubulin serve as loading controls. (D) The amounts of phosphorylated eIF2α and ATF4 were determined by densitometry quantitation of the film signal, normalised against total eIF2α and tubulin, respectively and expressed as indicated. *P < 0.1, **P < 0.01, ***P < 0.001, and ****P < 0.0001 (Student's *t*-test). The results are representative of more than 3 different experiments. **E.** RNA content was isolated and the amount of ATF4 mRNA relative to GAPDH mRNA was quantified by real-time q(RT)-PCR using the ΔΔCt method. **F.** Localisation of murine ATF4 mRNA in sorafenib-induced SGs. The indicated MEFs were fixed, permeabilised, and then incubated with 3 nM of an Alexa Fluor 488-labeled antisense RNA probe to detect ATF4 mRNA (right panels) or with the Alexa Fluor 488-labeled sense probe as control (left panels). Shown are typical results from five different fields and two different experiments containing a total of 1000 cells.

The above results showing a strong correlation between eIF2α phosphorylation and SGs induction raise the possibility that sorafenib-induced phosphorylation of eIF2α is responsible for SGs formation, which then suppresses P-eIF2α-mediated ATF4 upregulation. We first tested this hypothesis using MEFs. Dose-response experiments show that 25 μM of sorafenib is the minimum dose that induces SGs in a maximum (~ 30%) number of wild type (WT) MEFs (Fig. 3A–3B). This SGs formation in MEFs correlates with both the phosphorylation of eIF2α and the expression of its downstream ATF4 target (Fig. 3C). Sorafenib-induced SGs formation was completely blocked however in MEF eIF2α^S51A^, in which eIF2α Ser51 has been mutated to Ala [45] (Fig. 3A–3B). As expected, owing to the complete lack of eIF2α phosphorylation, no ATF4 expression was detected in eIF2α^S51A^ treated with sorafenib (Fig. 3C). These results confirm that phosphorylation of eIF2α is required for both SGs formation and ATF4 expression in MEFs treated with sorafenib.

### PERK is required for eIF2α phosphorylation and associated SGs formation in sorafenib-treated HCC

We then sought to determine which of eIF2α phosphorylating kinases is responsible for sorafenib-induced SGs. We first addressed this question using MEFs lacking either eIF2α phosphorylating kinase. As shown in Fig. 3C–3D, phosphorylation of eIF2α was efficiently (>2.5) induced in either WT MEFs or their counterpart MEFs lacking either PKR (PKR^−/−^) or GCN2 (GCN2^−/−^) (Fig. 3C–3D). This phosphorylation of eIF2α correlates with SGs formation, which occurs more efficiently in PKR^−/−^ (> 4) than in either WT or GCN2^−/−^ (2.5–3) (Fig. 3A–3B), excluding a major role of PKR in promoting sorafenib-induced SGs formation in MEFs. SGs formation occurring in sorafenib-treated GCN2^−/−^, was slightly less efficient than in WT (Fig. 3A–3B), suggesting that GCN2 may minimally contribute in promoting SGs formation in sorafenib-treated MEFs. We could not test the role of HRI in sorafenib-induced SGs formation in MEFs owing to the unavailability of HRI^−/−^ MEFs. Nevertheless, we found that SGs formation (Fig. 3A–3B) was dramatically reduced in sorafenib-treated MEFs lacking PERK (PERK^−/−^), as compared to sorafenib-treated WT MEFs. This is consistent with the reduced level of phosphorylation of eIF2α observed in sorafenib-treated PERK^−/−^ (Fig. 3C–3D). This residual phosphorylation of eIF2α detected in PERK^−/−^, which is likely due to either GCN2 or HRI, is however not sufficient to drive SGs formation. Thus, these results show that PERK is required for SGs formation in MEFs treated with sorafenib.

Surprisingly, and despite reduced level of eIF2α phosphorylation in sorafenib-treated PERK^−/−^, the expression of ATF4 was significantly (~ 5 fold) induced in this SGs-deficient cell line (Fig. 3C–3D). In contrary, under similar sorafenib treatment, ATF4 is barely induced (~ 1.5) in the highly SGs-forming PKR^−/−^ (Fig. 3C–3D), despite higher level induction of P-eIF2α (~ 4 fold) in these MEFs as compared to PERK^/−^ (~ 1.2 fold). The steady state level of ATF4 mRNA is however similar between PERK^−/−^ and its counterpart PKR^−/−^ under normal growth conditions and is slightly induced in both PKR^/−^ (~ 1.8 fold) and PERK^−/−^ (~ 2.2 fold) upon sorafenib treatment (Fig. 3E). This suggests that the differential expression of ATF4 observed between SGs-deficient PERK^−/−^ and SGs-forming PKR^−/−^ is largely translational, involving SGs. FISH experiments using specific oligos confirmed the localisation of ATF4 mRNA in SGs formed in both WT MEFs and PKR^−/−^ (Fig. 3F), corroborating the results obtained with HCC. In summary, MEFs studies revealed PERK as the main eIF2α phosphorylating kinase that triggers SGs formation upon sorafenib treatment. Those SGs contain a sub-fraction of repressed ATF4 mRNA, which may preclude its upregulation in sorafenib-treated MEFs.

**Figure 4:**
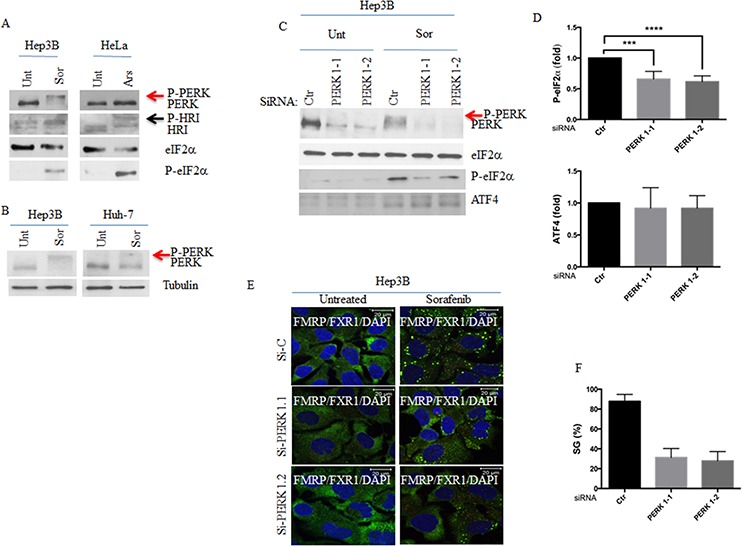
PERK activation is required for sorafenib-induced SGs **A.** Hep3B and HeLa were treated with sorafenib (10 μM) for 2 hours and with arsenite (150 μM) for 1 hour, respectively. Cells were collected and protein extracts analysed by western blot for the amounts of PERK, HRI and P-eIF2α. eIF2α serves as loading control. Red arrow denotes the supershifted migration of PERK in sorafenib-treated Hep3B indicating its activation. Activated HRI in arsenite-treated HeLa cells is indicated by a blue arrow. **B.** Hep3B and Huh-7 were treated with sorafenib (10 μM) for 2 hours. Cells were collected and protein extracts analysed by western blot for the activation of PERK as in (A). Red arrow denotes the supershifted migration of PERK in indicating its hyperactivation in Hep3B as compared to Huh-7, treated with sorafenib. **C–F.** Hep3B were treated with two specific PERK siRNAs for seventy-two hours and then incubated with sorafenib for two hours. (C) Cells were collected and protein content of collected cells was analysed by western blot for the expression of PERK, the phosphorylation of eIF2α and the amount of ATF4 using the corresponding antibodies. eIF2α serves as loading control. (D) The amounts of phosphorylated eIF2α and ATF4 were determined by densitometry quantitation of the film signal, normalised against total eIF2α and tubulin, respectively, and expressed as above. ***P < 0.001, and ****P < 0.0001 (Student's *t*-test). The results are representative of more than 3 different experiments. (E-F) Cells were processed for immunofluorescence to detect SGs using anti-FMRP and anti-FXR1 antibodies. DAPI stains nuclei. Shown in (E) are the merge pictures. Scale bars are shown. Representative results from 5 different fields and 5 different experiments containing a total of 1000 cells are shown. The percentage of cells harboring SGs (>3 granules/cell) is indicated in (F).

The results obtained using PERK^−/−^ prompted us to test if PERK is indeed required for eIF2α phosphorylation and associated SGs formation in sorafenib-treated Hep3B. PERK activation during stress occurs via its auto-phosphorylation [46, 47]. Due to its extensive phosphorylation, activated PERK migrates slower on SDS/PAGE than the inactive form [48]. This mode of activation was also reported for HRI, whose hyperphosphorylation during oxidative stress (e.g., arsenite treatment) retards its migration on SDS/PAGE [49]. We found that sorafenib treatment of Hep3B induces PERK activation as attested by its retarded migration in SDS-PAGE (Fig. 4A). Sorafenib was also shown to induce oxidative stress in HCC [50], which could thus trigger HRI activation. Control experiments show that arsenite activates HRI (but not PERK) as evidenced by its migration shift in SDS-PAGE (Fig. 4A). Sorafenib treatment of Hep3B does not activate HRI as demonstrated by the lack of its migration shift in SDS-PAGE (Fig. 4A). Together, the above results indicate that sorafenib treatment of HCC activates PERK but not HRI. PERK activation was also observed in sorafenib-treated Huh-7, albeit less prominent as compared with Hep3B (Fig. 4B). This minimal PERK activation in Huh-7 is consistent with the reduced level of both eIF2α phosphorylation (Figs. 2A–2B and S2B) and SGs formation (Figs 1A–1B and S2A) observed in Huh-7 as compared to Hep3B. These results further suggest that PERK is responsible for eIF2α phosphorylation and downstream SGs formation in HCC upon treatment with sorafenib.

To validate the above hypothesis, we either inactivated PERK using two recently developed specific PERK inhibitors (PERKi), [51, 52, 53, 54], or interfered with its expression using four specific PERK siRNAs. We found that treatment with either PERKi prevents sorafenib-induced PERK activation and significantly reduces the phosphorylation of its eIF2α substrate (Fig. S5A and data not shown). We obtained similar results by knocking-down PERK expression (Fig. 4C–4D and data not shown). Together, these results validate the role of PERK in phosphorylating eIF2α upon treatment of Hep3B with sorafenib. Both the inhibition of PERK activity with PERKi (Fig. S5A), or its depletion with siRNAs (Fig. 4E–4F) significantly (~ 35–40%) reduce the formation of SGs in sorafenib-treated Hep3B. Control experiments show that depletion of HRI does not affect significantly SGs formation in sorafenib-treated Hep3B (Fig. S6). Together, our results argue that PERK activation is required for SGs formation in sorafenib-treated Hep3B through eIF2α phosphorylation, corroborating MEFs results.

As mentioned above, we have found that a sub-fraction of the P-eIF2α-target ATF4 mRNA localises in SGs in both Hep3B and MEFs treated with sorafenib (Figs. 2H–2I and 3F). This localisation of ATF4 mRNA in SGs correlates with its lower expression in SGs-forming cells (Hep3B and PKR^−/−^), as compared to SGs-deficient cells (Huh-7 and PERK^−/−^). Thus, we considered the possible contribution of SGs in repressing ATF4 mRNA in sorafenib-treated HCC. We reasoned that if this is the case, disruption of SGs would rescue ATF4 upregulation in sorafenib-treated Hep3B. To test this possibility, we treated Hep3B with PERK siRNAs and assessed the expression of ATF4 following sorafenib exposure. We found that although depletion of PERK (Fig. 4C) reduces SGs formation (Fig. 4E–4F), it does not significantly promote ATF4 expression (Fig. 4C–4D). This result contrasts with those obtained using SGs-deficient PERK^−/−^, in which ATF4 expression was significantly induced as compared to SGs-forming PKR^−/−^ (Fig. 3C–3D). However unlike in PERK^−/−^, where the lack of PERK results in almost complete disruption of SGs formation (Fig. 3A–3B), depletion of PERK in Hep3B reduces but does not completely block SGs formation (Fig. 4E–4F). The lack of a clear positive effect on ATF4 expression due to PERK depletion in Hep3B could thus be due to the residual SGs that form in these cells, thereby repressing the associated ATF4 mRNA (see discussion). Despite the lack of direct evidence of the above assumption, our results showing the association of ATF4 mRNA with SGs combined to its weaker expression in SGs-forming cells, as compared to SGs-deficient cells, support a possible role of SGs in repressing at least a subfraction of ATF4 mRNA, thereby preventing ATF4 overproduction.

### Role of PERK-SGs axis in sorafenib resistance through modulation of ATF4 expression

**Figure 5:**
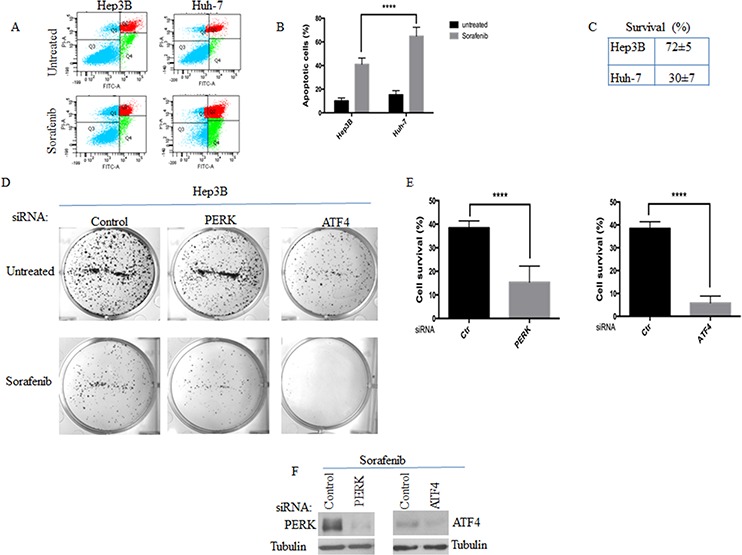
The activation of PERK-P-eIF2α-SGs pathway correlates with HCC resistance to sorafenib **A–C.** SGs-forming Hep3B are more resistant to sorafenib than SGs-deficient Huh-7. Hep3B and Huh-7 were treated with sorafenib (10 μM) for twenty four hours. (A-B) Cells were analysed by staining with annexin V-FITC and propidium iodide (PI) in flow cytometry. The percentage of apoptotic cells (right boxes) is indicated in (B) and is the means +/− s.e.m., from three independent experiments. ****P < 0.0001 (Student's *t*-test). (C) Clonogenic survival assays. Sorafenib-treated Hep3B were trypsinised, counted, replated in the absence of drug, and incubated for 10 days. Populations >20 cells were counted as one surviving colony. Data were calculated as the percentage of surviving colonies relative to the number found in control (untreated) plates. Results are expressed as the mean of triplicate measurements. ****P < 0.0001 (Student's *t*-test). **D–F.** Hep3B were treated with either control, or PERK, or ATF4 siRNAs for seventy-two hours. Cells were then incubated with sorafenib for twenty-four hours. The survival of Hep3B was assessed by the clonogenic assay as above. Representative results are shown in (D) with the indicated statistical calculation in (E). (F) SiRNA-treated Hep3B were collected following sorafenib treatment and their protein content was analysed by western blot to assess the depletion of both PERK and ATF4 using the corresponding antibodies.

The formation of SGs was shown to contribute to the resistance of cells to various stresses including radiation [1], oxidative stress [26] and proteasome inhibition [3, 10]. To determine if SGs formation correlates with HCC resistance to sorafenib, we treated both SGs-forming Hep3B and SGs-deficient Huh-7 with sorafenib and assessed cell death and survival using annexin V and clonogenic assays, respectively. We found that sorafenib induces cell death in both HCC (Fig. 5A–5B) and reduces their clonogenic survival (Fig. 5C). However, both cell death and loss of survival are significantly higher in SGs-deficient Huh-7, as compared to SGs-forming Hep3B (Fig. 5A–5C). Although correlative, these results suggest a contribution of SGs in cell resistance to sorafenib, which thus prompted us to test if reducing formation of SGs will further sensitise cells to sorafenib. To test this possibility, we first compared cell survival between SGs-deficient PERK^−/−^ and SGs-forming PKR^−/−^ upon treatment with sorafenib. These control experiments show that sorafenib induces a significant loss of clonogenic survival in PERK^−/−^, as compared to PKR^−/−^ upon sorafenib treatment (data not shown). We then tested if reducing SGs formation by depleting PERK affects Hep3B resistance to sorafenib. Control experiments show that depletion of PERK *per se* does not significantly induce apoptosis (data not shown) and slightly affect the survival in Hep3B (Fig. 5D). Surprisingly, depletion of PERK does not significantly promote apoptosis in sorafenib-treated Hep3B (data not shown). However, knocking down PERK expression with four specific siRNAs significantly abrogates Hep3B clonogenic survival to sorafenib (Figs. 5D–5F and data not shown), further supporting a role of PERK-SGs axis in driving sorafenib resistance.

SGs contribute to cell stress resistance either by regulating signaling pathways or affecting the expression of target mRNAs [55]. Our data described above support a role of SGs in buffering translation of ATF4 mRNA in sorafenib-resistant Hep3B, thereby keeping ATF4 expression minimal despite phosphorylation of eIF2α. Downregulation of ATF4 was shown to prevent resistance of cancer cells to anticancer drugs [21], indicating that a minimal expression of ATF4 is required for cancer cells survival. We thus asked if the minimal ATF4 expression, which is observed in SGs-forming Hep3B, is relevant for their resistance. Using two specific siRNAs, we found that depleting the ATF4 pool, which is still expressed in sorafenib-treated Hep3B, abrogates their survival (Fig. 5D–5F).

## DISCUSSION

Here we identified sorafenib as a potent inducer of SGs in HCC cells. While the formation of SGs was shown to inhibit cell death, their induction by chemotherapeutic drugs contributing to cancer cells resistance is still understudied. So far, two chemotherapeutic drugs, the proteasome inhibitor bortezomib (an FDA approved for the treatment of myeloma) and 5-fluorouracil (used to treat head, neck, breast, and colorectal cancers) have been described to induce SGs [4, 5, 10, 11]. Under both conditions, SGs induction requires the phosphorylation of eIF2α. While bortezomib induces phosphorylation of eIF2α via the activation of HRI, 5-fluorouracil triggers this modification by activating PKR. Here we identified PERK as the key eIF2α-phosphorylating kinase required for SGs induction upon treatment of HCC with sorafenib. This study also revealed a possible complex regulatory balance between SGs and P-eIF2α where SGs control the activation of the P-eIF2α-downstream ATF4 stress death pathway. Implication of PERK-eIF2α-SGs in HCC resistance to sorafenib is discussed.

Conflicting results of the effects of sorafenib on eIF2α phosphorylation have been reported. It was initially shown that treatment of human leukemia cells with sorafenib induces the phosphorylation of eIF2α, an event which was attributed to the induction of an ER stress [36]. More recent studies reported however that treatment of human urothelial cell lines with sorafenib inhibits eIF2α phosphorylation that is induced either by H_2_O_2_ or by doxorubicin [56]. While the origin of these opposite results is not known, it may indicate a cell-type or tissue-type specific effects of sorafenib. We found that sorafenib treatment of HCC efficiently induces eIF2α phosphorylation (Figs. 2A–2B, 4A–2B and S3B). This phosphorylation of eIF2α is most-likely responsible for the observed inhibition of translation initiation in sorafenib-treated HCC (Fig. 2C), although additional mechanisms might be involved. Nevertheless, our results showing that sorafenib inhibits translation initiation, which is one of the most promising chemotherapeutic target [57], further confirm this drug as a relevant chemotherapeutic drug. However, sorafenib-mediated inhibition of translation initiation occurs via phosphorylation of eIF2α; a modification which is known to promote cell survival in part by inducing SGs [4, 23, 24, 25].

Several evidences support the role of PERK-eIF2α phosphorylation in triggering SGs formation in sorafenib-treated HCC. First, we found that sorafenib treatment induces SGs in WT eIF2α fibroblast, but not in eIF2α^S51A^ (Fig. 3), in which eIF2α Ser51 has been mutated to Ala. Second, formation of SGs in HCC treated with sorafenib correlates with phosphorylation of eIF2α, which was significantly higher in SGs-forming Hep3B than in SGs-deficient Huh-7 (Figs. 2 and S2). Third, depletion experiments identified PERK as the main eIF2α phosphorylating kinase whose activation drives SGs induction in sorafenib-treated HCC (Fig. 4). We further corroborated these results using specific drugs inhibitors of PERK (Fig. S5) as well as in MEFs cells lacking this kinase (Fig. 3). Sorafenib-mediated activation of PERK in HCC is likely due to the induction of an ER stress, which is the main stress activator of PERK. This is also consistent with the study of Rahmani et al reporting the induction of ER stress and PERK activation in sorafenib-treated human leukemia (U937) cells [36]. It is however unknown whether sorafenib-induced PERK activation in U937 is sufficient to trigger SGs formation.

Our demonstration of the role of PERK in inducing phosphorylation of eIF2α and SGs formation in HCC does not exclude however subtle contribution of the other eIF2α kinases. With this respect, we noticed that both sorafenib-induced phosphorylation of eIF2α and SGs formation were reduced but not completely blocked, in PERK-depleted Hep3B (Fig. 4). It was previously reported that sorafenib induces oxidative stress [50], which is the main activator of the eIF2α kinase HRI [26]. Our depletion experiments make unlikely the possible contribution of HRI in the residual phosphorylation of eIF2α and associated SGs formation that are both detected in PERK-depleted HCC, upon sorafenib administration (Fig. S6). Whether PKR or GCN2 contribute to the phosphorylation of eIF2α and SGs formation that still occur in PERK-depleted HCC following treatment with sorafenib is still not clear. Nevertheless, our results clearly identified PERK as the major eIF2α kinase required for SGs formation in sorafenib-treated HCC.

With few exceptions [58], the formation of SGs is often associated with cell resistance to cytotoxic effects of stress. Our data support a possible contribution of SGs in HCC resistance to sorafenib. We found that sorafenib induces dramatic cell death by apoptosis in SGs-deficient Huh-7, as compared to SGs-forming Hep3B (Fig. 5A–5B), confirming previous studies [59]. Relative resistance to sorafenib-mediated apoptosis of SGs-forming Hep3B as compared to SGs-deficient Huh-7 is consistent with the well-established antiapoptotic role of SGs [60]. Massive apoptosis induction in SGs-deficient Huh-7 (Fig. 5A–5B) contrasts however with the results obtained upon partial disruption of SGs via PERK depletion in Hep3B (data not shown). The failure of PERK depletion to further sensitise Hep3B to sorafenib-induced apoptosis could be due to the residual antiapoptotic SGs that still form in Hep3B. Nevertheless, we consistently measured a significant negative effect of PERK depletion on the clonogenic survival of sorafenib-treated HCC (Fig. 5D–5F). This apparent apoptosis-independent loss of survival raises the possibility that PERK may antagonise sorafenib-induced non-apoptotic forms of cell death; an assumption that warrants additional studies for validation. Together, our data show that PERK activity is required for HCC resistance to sorafenib, in part by triggering the formation of SGs.

SGs-mediated repression of mRNAs encoding cell death functions is a valid survival sequestration mechanism by which SGs inhibit cell death pathways [1, 3, 4, 10, 11]. Here we identified ATF4 as a novel SGs-associated mRNA. The encoded ATF4 transcription factor is known to either activate or impede cell death pathways during stress. This dual role of ATF4 in cell death depends on both its level of expression and the type of stress applied. Our results show that ATF4 upregulation correlates with HCC sensitivity to sorafenib. While ATF4 mRNA was significantly expressed in the highly sorafenib-sensitive and SGs-deficient Huh-7, it was weakly expressed in the less sorafenib-sensitive and SGs-forming Hep3B (Fig. 2A–2B). This differential expression of ATF4 between SGs-forming Hep3B and SGs-deficient Huh-7 is unlikely to be transcriptional because ATF4 mRNA levels that are measured in the two cell lines are similar. Moreover, while ATF4 mRNA is found significantly enriched in the highly translating polysomes of sorafenib-treated Huh-7, it is largely excluded from polysomes in Hep3B treated with sorafenib (Fig. 2G). This result further indicates that the low expression of ATF4 mRNA in SGs-forming Hep3B (as compared to SGs-deficient Huh-7) is likely due to its translational repression. FISH experiments show a clear localisation of ATF4 mRNA in SGs that form in sorafenib-treated Hep3B (Fig. 2H). We obtained similar results in MEFs showing a strong inverse correlation between ATF4 expression and SGs formation where ATF4 mRNA localises. Because SGs can trap specific mRNAs repressing their translation, we attribute the repression of ATF4 mRNA translation that is observed in the highly SGs-forming cells (Hep3B and PKR^−/−^), at least partially to the association of a subfraction of its mRNA with sorafenib-induced SGs. Quantification of FISH experiments revealed that ~ 20% of ATF4 mRNA that is present in the cytoplasm of sorafenib-treated Hep3B is trapped in SGs (Fig. 2I). It should be noted however that most components of SGs so far analysed including mRNAs are highly dynamic as they rapidly (within secondes to few minutes) shuttle in and out SGs during stress [61]. Fluorescence recovery after photobleaching (FRAP) experiments investigating SGs dynamic further revealed that a significant (20–80%) fraction of individual SG-associated components is highly mobile [62], therefore escaping their detection in SGs in fixed cells using standard fluorescence techniques. In their study, Zhang et al [63] used an antisense 2′-*O*-methyl RNA probe to monitor by FRAP the dynamics of the association of endogenous cytoplasmic mRNAs with SGs induced in living COS cells by arsenite treatment. These FRAP experiments revealed that while ~ 30% of analysed mRNAs is diffusible, ~ 30% is rapidly moving in and out of SGs. The last 30% was found stably trapped in SGs, which is consistent with our quantification of FISH signal showing that approximately 20% of ATF4 mRNA is trapped in SGs. Our quantification of FISH signal does not take into account however any mobile fraction of ATF4 mRNA, which by transiently associating with SGs, are also kept untranslated. Future experiments investigating ATF4 mRNA shuttling activity in and out of SGs are thus required to circumvent FISH limitation and to elucidate if and how such shuttling activity contribute to ATF4 translation repression.

**Figure 6:**
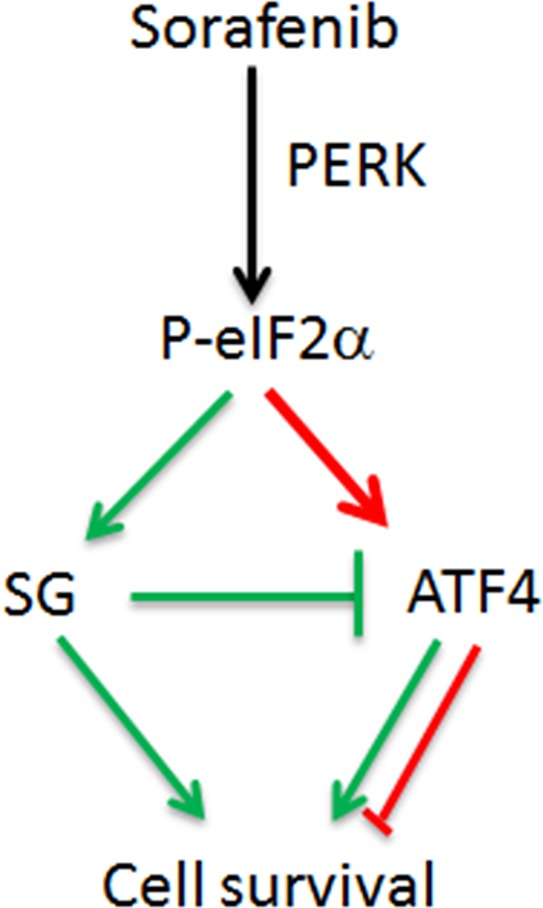
Model for the cross-talk between SGs and eIF2α phosphorylation in sorafenib-treated HCC In this model, sorafenib treatment of HCC activates PERK, which through phosphorylating eIF2α triggers both SGs formation and ATF4 expression. The association of ATF4 mRNA with SGs dampens however its overproduction, thereby contributing to HCC resistance to sorafenib.

Biological relevance of SGs in controlling cell homeostasis by repressing translation of specific mRNAs has been previously illustrated. For example, it was reported that in response to amino acid starvation, the translation of the 5′TOP transcripts encoding ribosomal and translation machinery are selectively repressed in SGs [29]. This limits the synthesis of unnecessary ribosomes, thereby adapting the cell metabolism to this nutritional type of stress. The selective repression of TOP mRNAs in SGs is mediated through their binding to the SGs components TIA/TIAR [29]. Similar mechanisms may thus account for the association of ATF4 mRNA with SGs induced by sorafenib, thereby limiting its expression. This minimal expression of ATF4 in mRNA SGs-forming HCC is nevertheless relevant since its elimination by siRNAs confers sensitivity of Hep3B to sorafenib (Fig. 5D–5F). Based on these results, we postulate a model (Fig. 6) where SGs may serve as a buffer that keeps ATF4 level sufficiently minimal to help their survival upon sorafenib treatment. Unfortunately, validation of this role of SGs in damping the expression of ATF4 in HCC cannot be conclusively provided by partially disrupting their formation through PERK depletion. Experiments designed to completely disrupt SGs, without affecting the phosphorylating of eIF2α, the upstream inducer of ATF4, are required to validate the role of SGs in regulating the expression of ATF4 mRNA in HCC. It is however noteworthy that although depletion of PERK reduces eIF2α phosphorylation, it does not reduce ATF4 expression in sorafenib-treated Hep3B (Fig. 4C–4D). We attribute this lack of negative effect of PERK depletion on reducing ATF4 expression to the availability of ATF4 mRNA for translation owing to the reduction of SGs level, thereby compensating at least partially the reduction of eIF2α phosphorylation. Clearly, further studies are required to determine how SGs regulate the expression of ATF4. Specifically, the identification of cis acting-RNA elements as well as trans-acting factors required for ATF4 mRNA association with SGs should contribute to define the mechanisms by which SGs repress ATF4 mRNA translation, thus affecting HCC resistance to sorafenib.

## MATERIALs AND METHODS

### Cell Lines and cultures

HeLa cervical cancer and MCF-7 breast cancer cells were purchased from the American Type Culture Collection (ATCC). LnCaP and PC3 prostate cancer cells were obtained from Dr. V. Fradette (Laval University). Huh-7 cells were provided by Dr. M. Santos (Université de Montréal) and Hep3B cells were received from Dr. M. Bilodeau (Université de Montréal). MEFs cells were previously described [4]. All cell lines were maintained in DMEM (Sigma, St. Louis, MO) supplemented with 10% fetal bovine serum (FBS; Sigma), penicillin, and streptomycin (Sigma).

### Drugs and drugs treatments

Sorafenib was purchased from Selleck Chemicals and dissolved in DMSO as a 10 mM stock solution, aliquoted and stored at −80°C. Sorafenib treatment was performed when cells had reached ~ 80% confluence. Before treatment, cells were cultured in DMEM supplemented with 5% FBS. PERK inhibitors, GSK2606414 and GSK2656157, were purchased from Selleck Chemicals and EMD Millipore, respectively. Both PERK inhibitors were dissolved in DMSO as a 10 mM stock solution, aliquoted and stored at −80°C. Cycloheximide and puromycin were purchased from Sigma (St Louis, MO) and dissolved in water as a 10 mg/mL and 25 mg/mL stock solutions, respectively, aliquoted and stored at −20°C.

### Antibodies

Phospho-specific anti-eIF2α and the pan anti-eIF2 were purchased from Cell Signaling Technology (Beverly, MA). Anti-FMRP, anti-FXR1, anti-eIF4E, anti-eIF4GI and anti-G3BP1 antibodies have been previously described [3]. Anti-RCK, anti-ATF4, and anti-HRI antibodies were purchased from Santa Cruz Biotech. Anti-PERK and anti-tubulin antibodies were obtained from Abcam. Anti-puromycin antibodies were obtained from EMD Millipore.

### Small-interfering RNA (siRNA) experiments and DNA transfection

All siRNAs were obtained from Dharmacon (Lafayette, CO). siRNA transfections were performed essentially as described, using Hiperfect reagent (Qiagen) following the manufacturer's protocol. Twenty-four hours before transfection, cells were trypsinized and plated at a density allowing to reach 50–60% confluence after twenty-four hours. For a 6-well plate, annealed duplexes were used at a final concentration of 10 nM. Forty-eight hours posttransfection, cells were treated with the same siRNA (5 nM) for additional forty-eight hours before treatment.

siRNA-PERK #1 : sense sequence : 5′-GGC AAU GAG AAG UGG AAU U-3′

siRNA-PERK #2 : sense sequence : 5′-GCUGAAAGAUGAAAGCACA-3′

siRNA-HRI #1 : sense sequence : 5′-GAT CTG AAG CCA AGA AAT A-3′

siRNA- HRI #2 : sense sequence : 5′-GGA AGA GGA CAG AGA GCA A-3′

siRNA-ATF4 #1 : sense sequence : 5′-CAG AUU GGA UGU UGG AGA A-3′

siRNA-ATF4 #2 : sense sequence : 5′-GCA AAG AGC UGG AAA AGA A-3′

For DNA transfection, Hep3B cells were transfected with a DNA vector encoding GFP-dcp1a in a 6-well plate using a Transfection reagent kit (Qiagen). At forty-eight hours later, cells were treated as indicated, fixed and processed for immunofluorescence.

### Immunofluorescence, poly(A)+ in situ hybridisation and RNA FISH

For immunofluorescence, all fixation, permeabilisation and staining procedures were done as we previously described [3].

For poly(A)^+^ mRNA *in situe* hybridisation studies, cells were first fixed in 3.7% paraformaldehyde for 20 min at room temperature, then permeabilised by a 15-min immersion in 0.1% Triton X-100/PBS. Poly(A)+mRNAs were detected using a custom made 59-tagged Alexa Fluor*H* 594-oligo [dT] (Invitrogen, Burlington, ON, Canada) diluted in PBS to a final concentration of 0.2 μM. Hybridisation was performed by incubating cells with the oligo (dT)/PBS for 30 minutes at 42°C, then overnight at 37°C. Cells were then washed twice with 2X SSC (30 min at 37°C) followed by two washes with 0.5X SSC (30 min at 37°C), and finally with PBS.

For FISH experiments, a DNA fragment encompassing the human ATF4 coding region was amplified by PCR using primers fused either with T3 (ATF4-forward: 5′-TCTCCGGGACAGATTGGATG-3′) or T7 (ATF4-reverse: 5′-GGAGGCTCCTATTTGGAGAG-3′) minimal promoter sequences. For MEFs, a DNA fragment encompassing the mouse ATF4 coding region was PCR-amplified using primers fused either with T3 (ATF4-forward: 5′-TCTCCGGGACAGATTGGATG-3′) or T7 (ATF4-reverse: 5′-GAGAAGGCAGATTGTCTGG-3′) minimal promoter sequences. The amplified fragments were used as a templates for *in vitro* transcription to produce either ATF4 antisense RNAs from the T7 promoter, or ATF4 sense RNAs from the T3 promoter, using the FISH Tag RNA Green Kit with Alexa Fluor 488 (Invitrogen, Burlington, ON, Canada), as we previously reported. Hybridisation of the probes was performed as described above. After hybridisation, cells were processed for immunofluorescence as above. RNA and proteins were visualised using the LSM 700 laser scanning confocal microscope (Zeiss), equipped with a Zen software for images acquisition and processing. Images were acquired using the following settings: 63X oil objective, 0.06 um for pixel size, and 1.00 airy units as pinhole.

### Quantitative RT-PCR

RNA was extracted with Trizol reagent (Life Technology). RNA was resuspended in water and analysed by qRT-PCR. RT reactions were performed using the Quantitect Reverse Transcriptase kit (Qiagen). Each reaction contained 2 μl of RNA (isolated using the RNeasy Plus Mini Kit; Qiagen) at 100 ng/ml, 10 μl of RNase-free water, 2 μl of genomic DNA Wipeout Buffer 7X, 4 μl of Quantiscript RT Buffer 5X, 1 μl of RT Primer Mix and 1 μl of Quantiscript Reverse Transcriptase. Real-time PCR reactions were prepared using the Power SYBRH Green PCR Master mix (Applied Biosystems, Streetsville, ON, Canada) in a total volume of 25 μl: 12.5 μl of PCR Master Mix, 0.67 μl of forward primer at 3.75 μM, 0.67 μl of reverse primer at 3.75 μM, 9.2 μl of deionized (Milli-Q grade) water and 2 μl of RT-PCR. Reactions were run and data then analysed using the MX3000 Real-Time PCR system (Applied Biosystems) as described [10]. To prepare templates for the ATF4 mRNA, the oligonucleotide pair used was: 5′-CACTAGGTACCGCCAGAAGA-3′ (forward), and 5′-AATCCGCCCTCTCTTTTAGA-3′ (reverse). For preparing templates corresponding to the Actin mRNA, the oligonucleotide pair used was: 5′-GGAAATCGTGCGTGACATT-3′ (forward), and 5′-CTAGAAGCATTTGCGGTG-3′ (reverse). Templates for the murine ATF4 mRNA were prepared using the following oligonucleotide pair: 5′-ACATTCTTGCAGCCTTTCCC-3′ (forward), and 5′-TAAGCAGCAGAGTCAGGCTT-3′ (reverse). To prepare templates for the murine GAPDH mRNA, we used 5′-AACGACCCCTTCATTGACCT-3′ (forward), and 5′-TGGAAGATGGTGATGGGCTT-3′ (reverse) as oligonucleotides.

### Polysomal profiles and analyses of polysomal-associated RNA

Polysomal profiles were performed as follow: HCC cells were grown in 100-mm tissue culture dishes to ~ 80% confluence, treated with sorafenib, then scrapped in 1 ml of polysomal buffer (20 mM Tris, pH 7.5, 150 mM NaCl, 1.25 mM MgCl_2_, 5 U/ml RNAsine [GE Healthcare], EDTA-free protease inhibitor cocktail [Complete; Roche, Indianapolis, IN], and 1 mM dithiothreitol), and Nonidet P-40 was added to a final concentration of 1% for lysis of 15 min on ice. Extracts were clarified by centrifugation at 12,000 *g* for 20 min at 4°C. Cytoplasmic extracts were loaded on each 15–55% (w/v) linear sucrose gradient and further fractionated and analysed as described previously [64]. For RNA analysis, fractions corresponding to monosomes, light and heavy polysomes were collected and their RNA content precipitated. Following phenol extraction and precipitation, RNA was resuspended in water and prepared for qRT-PCR as described above.

### [^35^S]Methionine labeling and ribopuromycylation

For metabolic labeling, cells in 6-well plates were labeled for 60 min with 1 ml methionine-free DMEM (Sigma) supplemented with 10% fetal bovine serum and 50 μCi/ml of [^35^S] methionine (Easy Tag, PerkinElmer/NEN Radiochemicals). For puromycylation, cells were labelled with 10 μg/ml of puromycin for 10 minutes. Puromycylated nascent polypeptides chains released from ribosomes were detected by western blotting using anti-puromycin antibodies as previously described [43].

### Annexin V-fluorescein isothiocyanate/propidium iodide assay and clonogenic survival assays

For annexin V, adherent and detached cells were collected, washed with ice-cold PBS, and resuspended in ice-cold binding buffer (10 mM HEPES/NaOH, pH 7.4, 140 mM NaCl, 2.5 mM CaCl_2_). Following staining with annexin V-fluorescein isothiocyanate, and propidium iodide, cells were counted, and dead cells were examined by flow cytometry. For clonogenic survival assays, cells were washed with PBS, trypsinized, counted, replated in 6-well plates at 1000 cells/well in the absence of drug, and incubated for 7–10 days. Before colony counting, cells were washed with PBS, stained (0.1% (w/v) crystal violet in a 0.0037% (v/v) formaldehyde solution in PBS), rinsed, and dried. Isolated colonies were counted.

## SUPPLEMENTARY FIGURES


