# Supplementary material for: Sorafenib, a multikinase inhibitor, induces formation of stress granules in hepatocarcinoma cells
Source: Oncotarget. 2015 Nov 2;6(41):43927–43. doi: 10.18632/oncotarget.5980 (PMC4791277; doi:10.18632/oncotarget.5980)
Supplement: Supplementary file 1 [file oncotarget-06-43927-s001.pdf]

SUPPLEMENTARY FIGURES

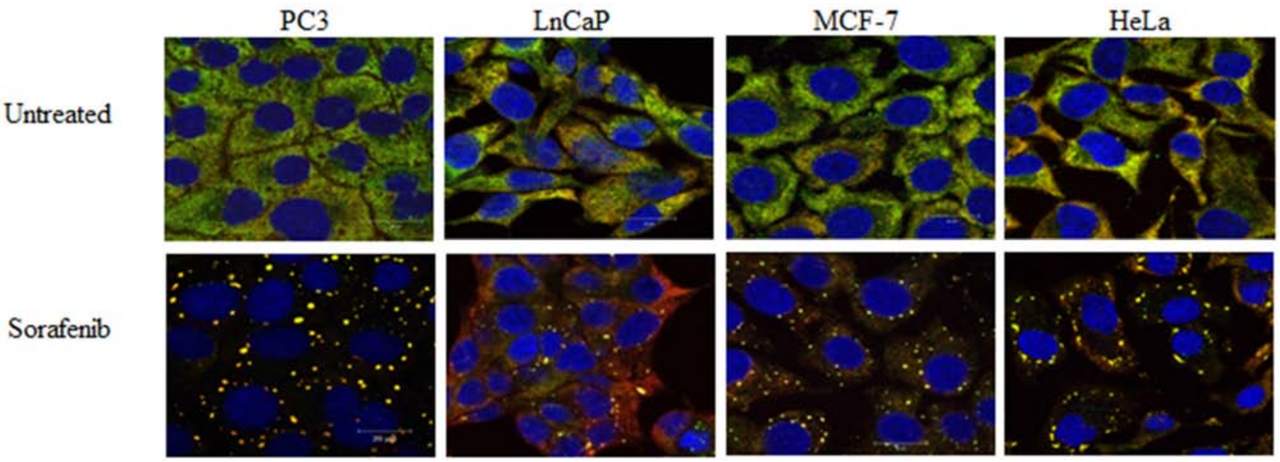

Supplementary Figure S1: Sorafenib induces SGs in prostate, breast and cervix cancer cells. LnCaP and PC3 (prostate), MCF-7 (breast), and HeLa (cervix) cancer cells were treated with sorafenib (20 μM) for two hours or left untreated. Cells were processed for immunofluorescence as described in Figure 1. Blue DAPI staining depicts nuclei.

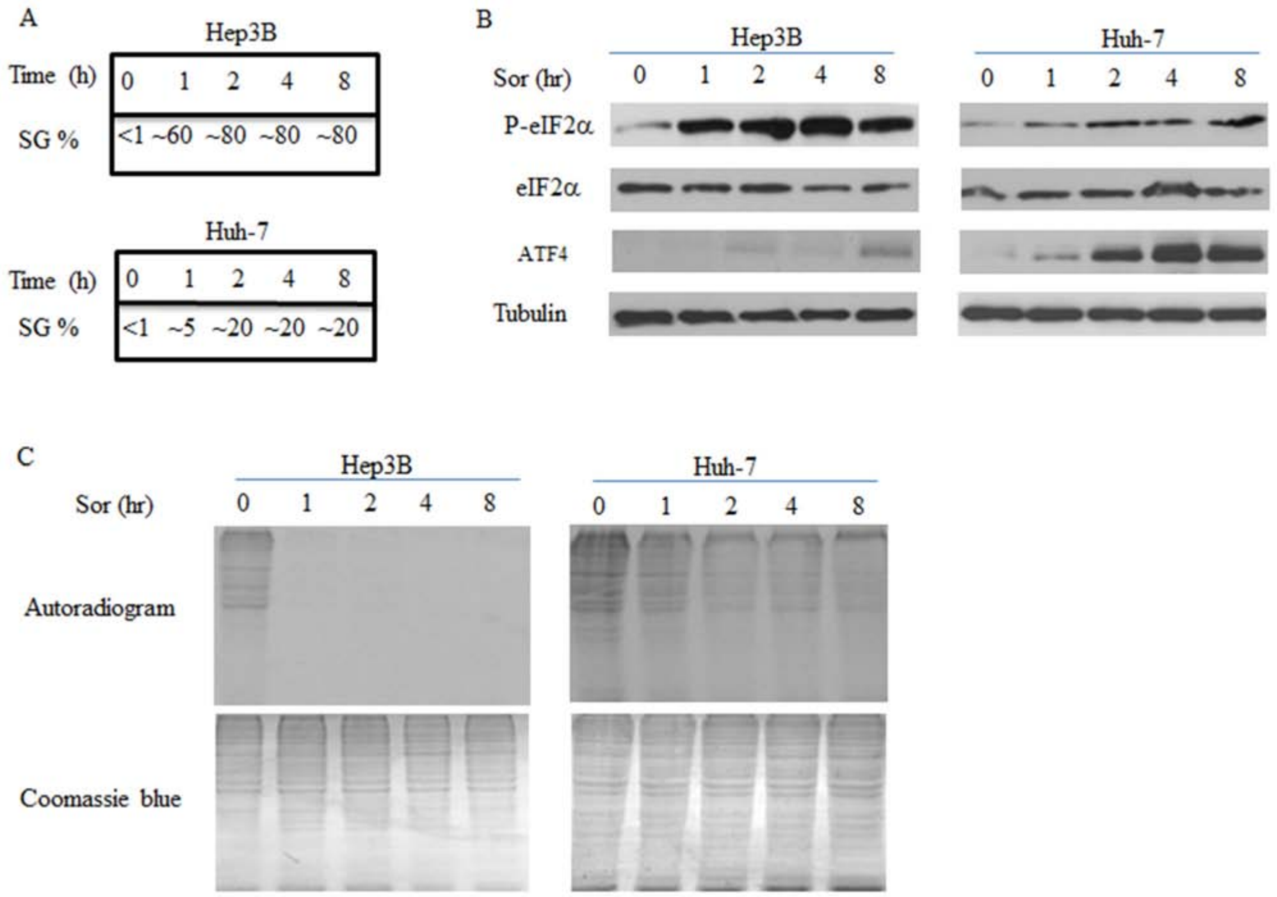

Supplementary Figure S2: Time course analysis of formation of SGs, phosphorylation of eIF2α, ATF4 expression and general translation in sorafenib-treated HCC. Hep3B and Huh-7 cells were treated with sorafenib for the indicated time points. **A.** Cells were processed for immunofluorescence to detect SGs using anti-FMRP and anti-G3BP1 antibodies. DAPI stains nuclei. The indicated percentage of cells harboring SGs was calculated as described in Figure 1. **B.** Cells were collected and their content was analysed for the expression of phospho-eIF2α and ATF4 by western blot using specific antibodies. Tubulin and eIF2α serve as loading controls. **C.** One hour following sorafenib addition, cells were incubated for an additional hour with [^{35}S] methionine (50 µCi/ml) in presence of sorafenib. Proteins were extracted, resolved on SDS-polyacrylamide gels, stained with Coomassie Blue (*bottom* panel), and detected by autoradiography (*top* panel).

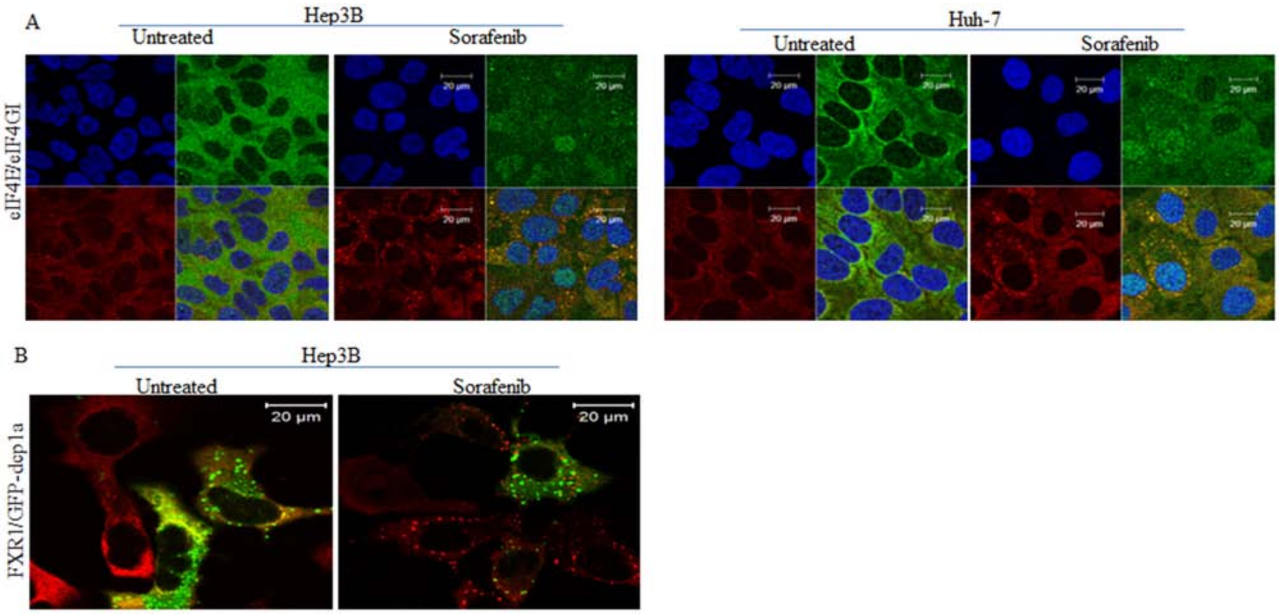

Supplementary Figure S3: Sorafenib-induced SGs in HCC contain eIF4E and eIF4GI but lack the GFP-dcp1a, a classical P-bodies marker. A. Hep3B and Huh-7 cells were treated with sorafenib (10 μM) for two hours or left untreated. Cells were processed for immunofluorescence to detect SGs using antibodies specific to eIF4E and eIF4GI. Blue DAPI staining depicts nuclei. Scale bars are shown. **B.** Hep3B cells were transfected with pEGFP plasmid encoding GFP-dcp1a. Forty-eight hours later, cells were treated with sorafenib (10 μM) for two hours then processed for immunofluorescence to detect SGs (in red) using anti-FXR1 antibodies. Green signal corresponds to GFP-dcp1a detected in P-bodies.

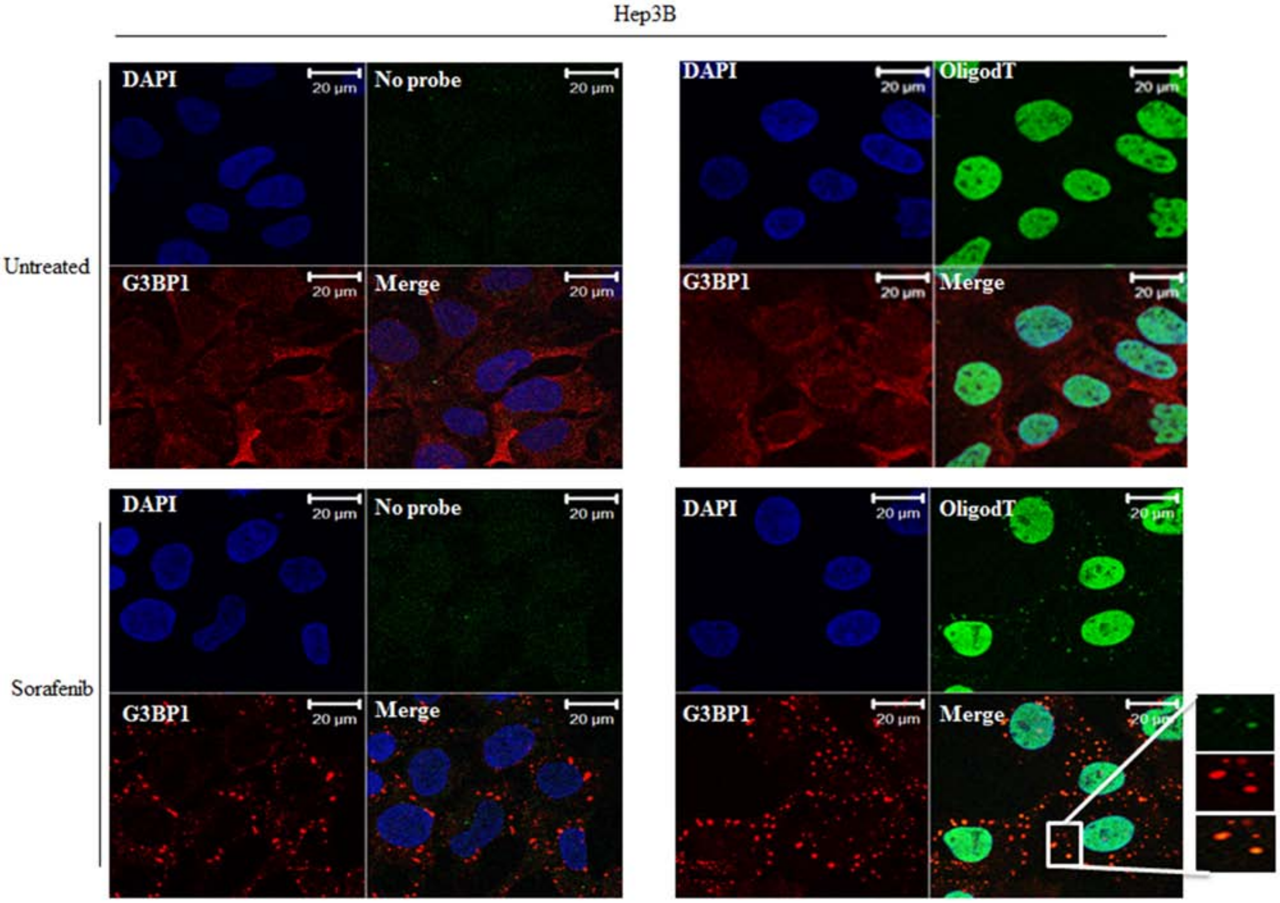

Supplementary Figure S4: Sorafenib-induced SGs contain poly(A)$^{+}$ mRNA. Hep3B cells were treated with 10 µM sorafenib for 2 hours, fixed, permeabilised, and incubated with 0.2 µM of an Alexa Fluor 488-labeled oligo(dT) probe to detect poly(A)$^{+}$ mRNA (green signal). SGs were detected using anti-G3BP1 antibodies (red signal). Representative results from 5 different fields and 2 different experiments containing a total of 500 cells are shown. Scale bars are shown. Blue staining is for nuclei.

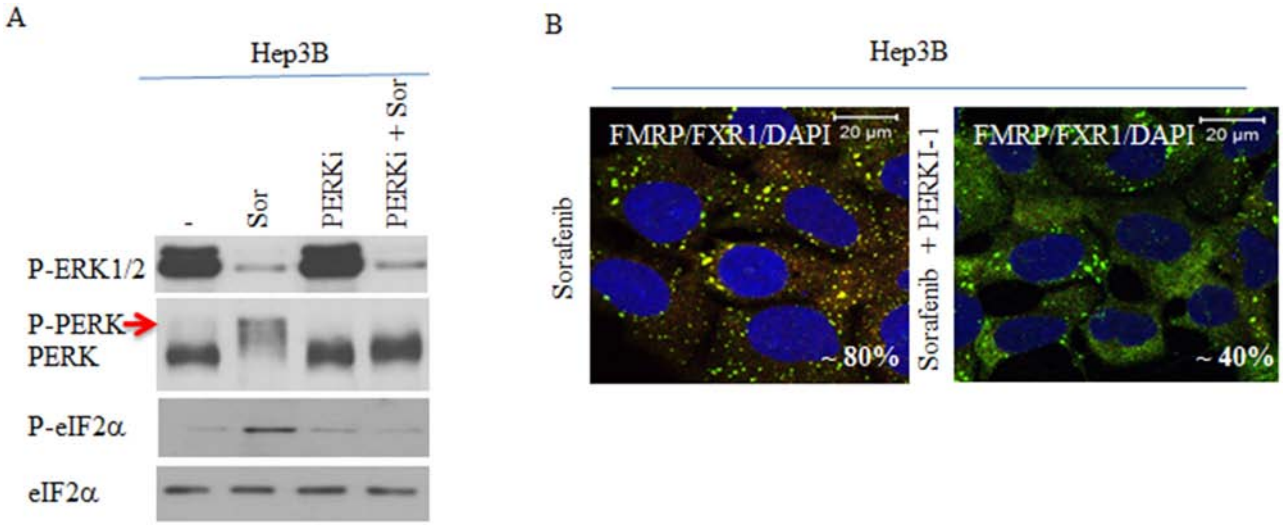

Supplementary Figure S5: Pharmacological inactivation of PERK reduces SGs formation in sorafenib-treated Hep3B. A–B. Hep3B cells were preincubated with 100 nM PERK inhibitors (PERKi) for two hours. Sorafenib (10 μM) and PERKi (GSK2606414; 100 nM) were then added for another two hours. (A) Cells were lysed and protein content was analysed by western blot for the activation of PERK and the phosphorylation of both eIF2α and ERK using the corresponding antibodies. eIF2α serves as a loading control. We obtained similar results using a second PERKi, GSK2656157. (B) Cells were collected, processed for immunofluorescence to detect SGs and the number of SGs-positive cells was scored as in Figure 1.

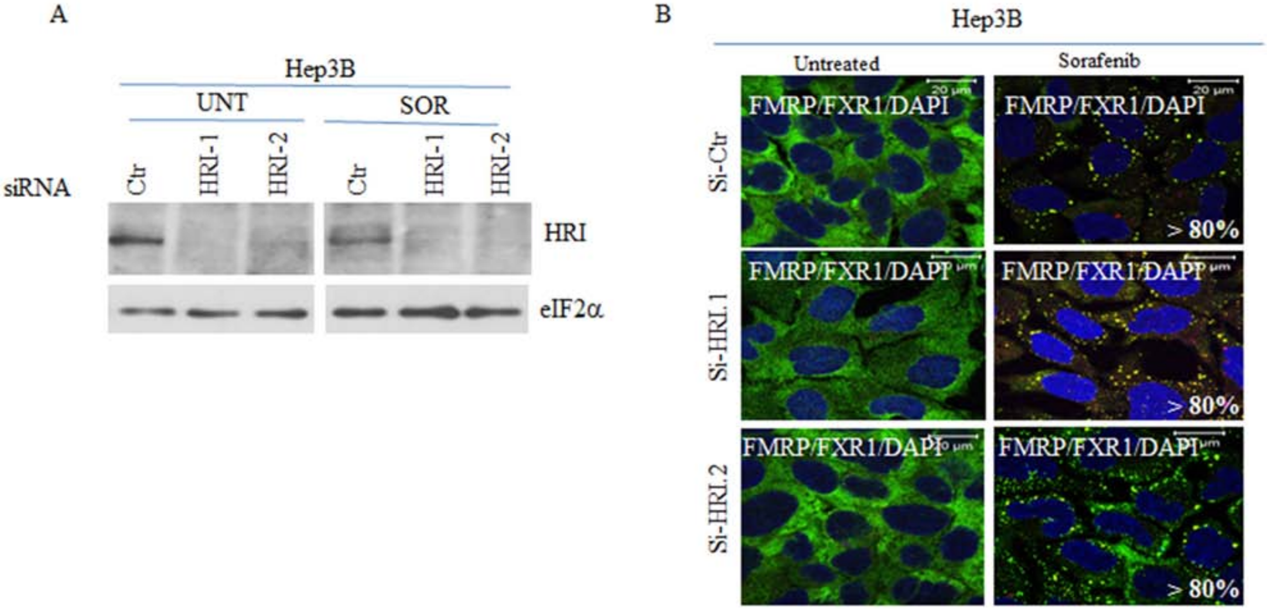

Supplementary Figure S6: Depletion of HRI does not affect SGs formation in sorafenib-treated Hep3B. A–B. Hep3B were treated with two specific HRI siRNAs for seventy-two hours then incubated with sorafenib for two hours. (A) Cells were collected and protein content was analysed by western blot for the expression of HRI and for the phosphorylation of eIF2α using the corresponding antibodies. eIF2α serves as loading control. (B) Cells were processed for immunofluorescence to detect SGs using anti-FMRP and anti-FXR1 antibodies. DAPI stains nuclei. Shown are merge pictures. The indicated percentage of cells harboring SGs was calculated as in Figure 1. Scale bars are shown.
